# Neonatal Epidermolytic Ichthyosis Caused by a KRT10 Mutation (c.467G>A, p.Arg156His): A Case Report

**DOI:** 10.1002/ccr3.70682

**Published:** 2025-07-29

**Authors:** Elke Smits, Gunnar Naulaers, Maria C. Bolling, Eric Legius, Caroline Colmant

**Affiliations:** ^1^ Department of Pediatrics University Hospitals Leuven Leuven Belgium; ^2^ Department of Development and Regeneration KU Leuven Leuven Belgium; ^3^ Neonatal Intensive Care Unit University Hospitals Leuven Leuven Belgium; ^4^ Department of Dermatology University Medical Center, University of Groningen Groningen the Netherlands; ^5^ Department of Human Genetics KU Leuven and University Hospital Leuven Leuven Belgium; ^6^ Department of Dermatology University Hospitals Leuven Leuven Belgium

**Keywords:** blistering skin disease, congenital erythroderma, epidermolysis bullosa, epidermolytic hyperkeratosis, epidermolytic ichthyosis

## Abstract

We present a neonatal case of skin blisters and erythema. While epidermolysis bullosa was initially suspected, immunofluorescence antigen mapping and genetic testing confirmed epidermolytic ichthyosis, with a heterozygous pathogenic variant in the *KRT10* gene (c.467G>A, p.Arg156His). A multidisciplinary approach is essential for accurate diagnosis and treatment of neonatal blistering conditions.

## 1 Introduction

Epidermolytic hyperkeratosis or epidermolytic ichthyosis (EI, OMIM 113800) is an uncommon keratinization disorder which affects approximately 1:200000 infants and is caused by pathogenic heterozygous variants in the genes *KRT1* and *KRT10* [1]. The clinical phenotype is defined by congenital erythroderma, superficial erosions, and epidermal blisters, evolving towards thickening of the epidermis (progressive hyperkeratosis) later on in life [2]. By losing the protective barrier of the skin, these newborns are at risk of dehydration, electrolyte disturbances, and severe infections such as sepsis [3]. Presentation at birth can be confused with epidermolysis bullosa (EB), as it may present with blistering and skin detachment in the neonatal period. EI shows an autosomal dominant inheritance pattern with complete penetrance. Half of the cases are caused by de novo mutations, which affect both sexes equally [4]. Symptom reduction should be the main core of treatment in a multidisciplinary approach, as there exists no cure [5]. We present a case of a male newborn where EB was initially suspected, but genetic testing confirmed EI, identifying a pathogenic heterozygous variant in the *KRT10* gene (c.467G>A, p.Arg156His).

## 2 Case History

**FIGURE 1:**
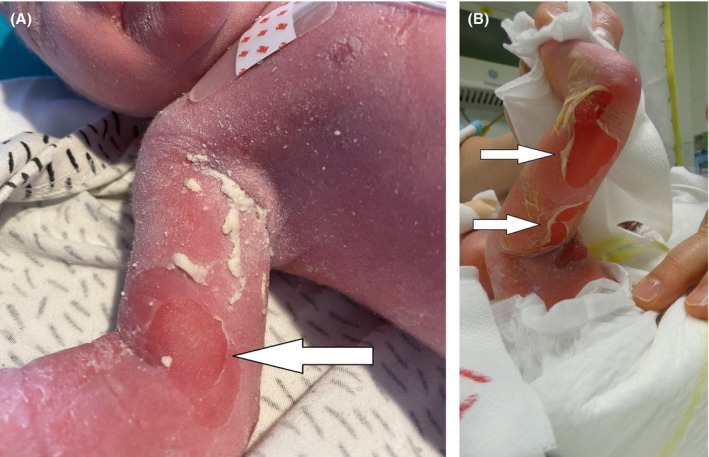
Clinical features in the affected newborn: (A) The patient's right upper limb showing diffuse erythroderma and areas of denuded skin (arrow), and (B) superficial blister formation (arrows) on the flexural surface of the lower limb.

**FIGURE 2:**
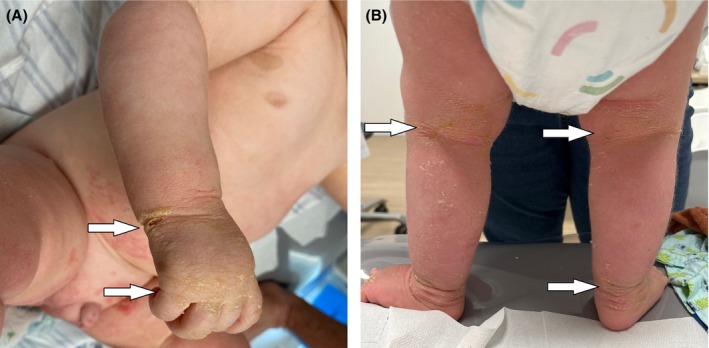
Clinical features showing the characteristic thickening, scaling, and yellowish appearance of the skin (arrows): (A) on the hand, and (B) on the flexural surfaces of the lower extremities, most notably in the popliteal folds and around the ankles.

A male, Caucasian newborn was transferred to the EB referral center at the University Hospital of Leuven after birth due to the immediate presentation of blistering and erythema of the skin. He was the second child of non‐consanguineous healthy parents, without any familial background of skin disorders. After induction for maternal pre‐eclampsia, he was born at a postmenstrual age of 36 weeks and 3 days with vacuum delivery. The birthweight was 2310 g (10th percentile) and birth length was 45 cm (14th percentile). Upon admission, physical examination revealed a neonate with generalized erythroderma, blisters, and denuded areas of skin, particularly at flexural surfaces and on the hands (Figure 1). There was clinically no involvement of the nails, mucosal surfaces, or hair, and no other extracutaneous abnormalities were noted. He was placed on cotton blankets to minimize shearing forces and cared for following the newborn EB protocol by a specialized care team. By the second day of life (DOL) oliguria was observed and persisted despite optimization of intravenous fluid management and discontinuation of nephrotoxic medication (ibuprofen). Intravenous furosemide (DOL 4–7) at a maximum dose of 5 mg/kg/day did not improve urine output. However, intravenous bumetanide (DOL 8–16), administered at a maximum dose of 0.1 mg/kg four times daily, successfully normalized diuresis. Loop diuretics were discontinued on DOL 16. A respiratory decline was noted on DOL 6. Non‐invasive respiratory therapy with high flow nasal cannula was initiated to address an obstructive breathing pattern. However, within 24 h, intubation and mechanical ventilation became necessary due to respiratory failure characterized by CO_2_ retention and increased oxygen requirements (maximum FiO_2_ of 50%). There were no clinical, radiological, or laboratory findings suggestive of pneumonia. Respiratory support was successfully discontinued on DOL 18 after diuresis had normalized and pulmonary edema caused by fluid overload had resolved. Finally, pancytopenia (platelet count 57,000/L, red blood cell count 2300/L, white blood cell count 2460/L) was observed around DOL 6. A single transfusion of packed red blood cells and platelets (15 mL/kg each) was administered on DOL 8 due to anemia (hemoglobin level of 8 g/dL) and the presence of mildly bloody endotracheal secretions, despite normal coagulation parameters. A spontaneous resolution of pancytopenia was observed by DOL 14; however, its etiology remains unclear. The patient was transferred to a local hospital on DOL 21. At that time, he received partial parenteral nutrition, accounting for 45% of total daily fluid intake. Enteral feeding at 90 mL/kg/day was administered via nasogastric tube, with good gastrointestinal tolerance. No systematic analgesics or other medication were given at the time of discharge. He continued follow‐up appointments at our institution by the multidisciplinary EB team, including a dermatologist, pediatrician, EB nurse, and dietitian. Re‐evaluation at 5 months of age showed features of transition from a bullous to an ichthyotic disease. Elbows and knees showed hyperkeratotic plaques, although there were still recurrent erosions on the back and legs. Xerosis cutis and pruritis were present. At 8 months of age, he was hospitalized for intravenous antibiotics (flucloxacillin 100 mg/kg/day for 48 h) for a bacterial infection of the skin, concomitant with a viral upper respiratory tract infection. Wound cultures showed *Streptococcus pyogenes* and *Staphylococcus aureus*. At the most recent follow‐up visit (age 14 months), the clinical aspect consisted mostly of xerosis and hyperkeratosis of the flexural areas and legs, along with some erosions on the arms and diffuse erythema (Figure 2). Pruritis remains a major problem causing heavy sleep loss. The child's weight initially dropped below the 3rd percentile but recovered well, reaching the 25th percentile by the age of 1 year. Growth remained narrowly below the 3rd percentile at that time. During the follow‐up period, he achieved all developmental milestones within the expected timeframes.

## 3 Differential Diagnosis and Investigations

**FIGURE 3:**
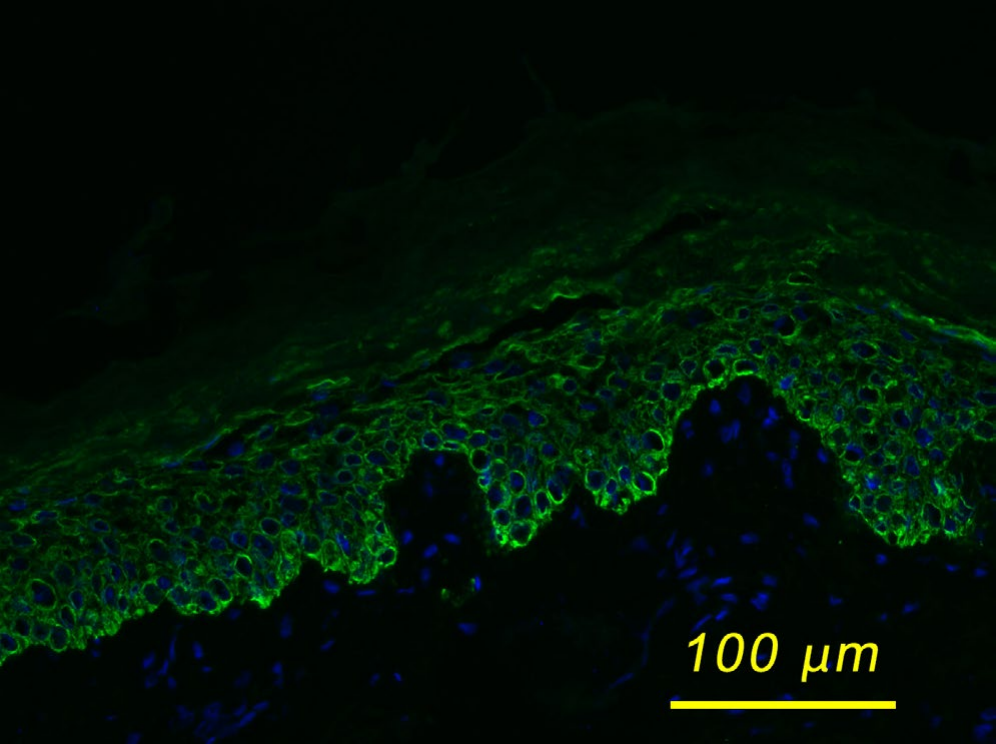
Immunofluorescence staining of the skin biopsy specimen obtained from a clinically affected area (lower left limb), using the LL001 antibody against *KRT14*, which highlights the basal keratinocytes. A distinct subcorneal cleft is observed, consistent with suprabasal epidermal fragility resulting from the disrupted keratin filament architecture in EI. Image acquired at 20× magnification.

A skin biopsy was taken after birth and sent to the UMCG Center of Expertise for Blistering Diseases in Groningen, the Netherlands, for further classification as EB was initially suspected. Histopathological examination revealed splitting of the stratum granulosum caused by acantholysis. Immunofluorescence staining with the LL001 antibody targeting keratin 14 (*KRT14*), a marker of basal keratinocytes, demonstrated subcorneal cleft formation (Figure 3). These findings, together with further histopathological investigations, were suggestive of EI. A diagnostic workup for severe combined immunodeficiency (SCID) and congenital/neonatal infections—including herpes simplex, syphilis, staphylococcal scalded skin syndrome (SSSS)—was negative. Congenital erythropoietic porphyria (CEP) was also excluded, based on measurements of porphyrin in plasma and urine, as the skin erosions were accompanied by thrombocytopenia, leukopenia, and hemolytic anemia. On the other hand, there was never overexposure to light, and no facial hypertrichosis or hypersplenism was present. Peripheral blood was obtained for genetic analysis.

## 4 Conclusion and Results

An exome sequencing panel for congenital blistering skin diseases revealed a heterozygote pathogenic variant (c.467G>A, p.Arg156His) in *KRT10*, which encodes keratin 10. This amino acid change on a highly conserved position affects the *KRT10* protein function and has been previously reported in cases of EI [6, 7, 8, 9]. This confirmed the diagnosis of Epidermolytic Ichthyosis in our patient. Sanger sequencing performed for carrier detection in both parents yielded negative results, suggesting a de novo mutation. Gonadal mosaicism in one of the parents cannot be excluded.

EI is a rare genetic disease presenting with blistering at birth and later developing ichthyosis and hyperkeratosis. Diagnosis at birth can be difficult due to overlapping symptoms with EB. While skin biopsy and immunofluorescence may help exclude EB, genetic testing is essential to confirm the definitive diagnosis. A multidisciplinary approach in a congenital blistering skin disease reference center is mandatory.

## 5 Discussion

EI is caused predominantly by pathogenic variants in the highly conserved regions of keratin 1 (*KRT1*) and keratin 10 (*KRT10*) [10, 11]. These keratins are essential elements of the cytoskeleton and are found in the suprabasal layers of keratinized stratified epithelial tissue. A defect in a section of this matrix may disrupt epidermal stability, resulting initially in mainly blistering and erosions [12, 13]. *KRT1* and *KRT10* also play a role in the inhibition of cell proliferation. This explains the emergence of hyperkeratosis in the latter stage of EI, as an insufficient inhibition of proliferation and the constant stimuli of cytokine release during cell rupture [14]. As the underlying mutation is located in keratin genes, this condition is classified within keratinopathic ichthyoses (KPI), a rare group of inherited non‐syndromic ichthyoses. KPI encompasses a spectrum of clinical phenotypes with varying severity. Mutations in *KRT1* or *KRT10* are associated with EI, but also contribute to related disorders such as ichthyosis Curth‐Macklin, annular epidermolytic ichthyosis, ichthyosis with confetti, autosomal recessive and nevoid forms. Mutations in *KRT2* result in a more superficial epidermal blistering pattern, termed superficial epidermolytic ichthyosis (SEI), recognized as the second major type of KPI [15]. The genotype–phenotype correlation within KPI is notably complex. Mutations in different genes can result in overlapping clinical presentations, while mutations within the same gene may lead to diverse phenotypes, as illustrated in Table 1 [16, 17]. This complexity makes the differential diagnosis of these diseases difficult, particularly at birth.

**TABLE 1:** Published cases of the KRT10‐R156H mutation (c.467G>A (p.Arg156His)), including associated phenotypic and genotypic findings.

Gender	Age	Origin	Pattern of EI	Blisters (marked location)	Erythroderma	Hyperkeratosis (marked location)	PPK	Inheritance	Missense mutation R156H	Domain	Reference
Female	NM	NM	Generalized	Present	NM	Present (flexural)	NM	NM	Single heterozygous point	1A	Cheng J et al., *Cell* (1992)
Female	NM	NM	Generalized	Present	NM	Present (flexural)	NM	Familial (child of 1)	Single heterozygous point	1A	Cheng J et al., *Cell* (1992)
Female	NM	NM	Generalized	Present	Present	Present (flexural)	NM	Familial^a^ (child of 15)	Single heterozygous point	1A	Cheng J et al., *Cell* (1992)
Female	NM	NM	Generalized	Present	Present	Present (flexural)	NM	Familial (child of 3)	Single heterozygous point	1A	Cheng J et al., *Cell* (1992)
Female	17Y	NM	Generalized	Present	NM	Extensive (limbs, trunk, face)	No	Sporadic	Single heterozygous point	1A	Rothnagel JA et al., *Science* (1992)
NM	NM	NM	Generalized	Very mild	NM	Present (dorsum feet, limbs, neck)	No	Sporadic	Single heterozygous point	1A	Rothnagel JA et al., *Hum Mol Genet* (1993)
Female	NM	NM	Generalized	NM	NM	Present (scalp, trunk, limbs)	No	Sporadic	Single heterozygous point	1A	Rothnagel JA et al., *Hum Mol Genet* (1993)
Male	NM	NM	Generalized	NM	NM	Present (scalp, trunk, limbs)	no	Familial (child of 7)	Single heterozygous point	1A	Rothnagel JA et al., *Hum Mol Genet* (1993)
Male	NM	NM	Generalized	NM	NM	Present (scalp, trunk, limbs)	No	Familial (sibling of 8)	Single heterozygous point	1A	Rothnagel JA et al., *Hum Mol Genet* (1993)
Female	NM	NM	Generalized	NM	NM	Present (scalp, trunk, limbs)	No	Familial (child of 9)	Single heterozygous point	1A	Rothnagel JA et al., *Hum Mol Genet* (1993)
Female	NM	NM	Generalized	NM	NM	Present (scalp, trunk, limbs)	No	Familial (child of 10)	Single heterozygous point	1A	Rothnagel JA et al., *Hum Mol Genet* (1993)
Female	21Y	NM	Generalized	Present	Present	Extensive (flexures)	No	Sporadic	Single heterozygous point	1A	Chipev C et al., *Am J Hum Genet* (1994)
Female	NM	NM	Generalized	Mild	No	Mild (joints)	No	Sporadic	Single heterozygous point	1A	Chipev C et al., *Am J Hum Genet* (1994)
Female	NM	NM	Generalized	Mild	No	Mild (joints)	No	Familial (child of 13)	Single heterozygous point	1A	Chipev C et al., *Am J Hum Genet* (1994)
Female	NM	NM	EN	NM	NM	Extensive streaks (lines of Blaschko)	NM	Sporadic	Single heterozygous point	1A	Paller A et al., *N Engl J Med* (1994)
Female	5Y	Japanese	Generalized	Present	Present	Extensive (neck, joints, trunk)	No	Sporadic	Single heterozygous point	1A	Nomura K et al., *Jpn J Hum Genet* (1997)
Male	10Y	Korean	Generalized	Present	Present	NM	No	Sporadic	Single heterozygous point	1A	Yang JM et al., *J Detmatol Sci* (1999)
Female	NM	British	Generalized	Present	Present	Present	NM	Familial	Single heterozygous point	1A	McLean WHI et al., *Exp Dermatol* (1999)
Male	NM	British	Generalized	Present	Present	Present	NM	Familial (child of 18)	Single heterozygous point	1A	McLean WHI et al., *Exp Dermatol* (1999)
Female	NM	Japanese	Generalized	Present	Present	Present	NM	Sporadic	Single heterozygous point	1A	Mayuzumi N et al., *J Eur Acad Dermatol Venereol* (2000)
Male	NM	Japanese	Generalized	Present	Present	Present	NM	Familial (child of 20)	Single heterozygous point	1A	Mayuzumi N et al., *J Eur Acad Dermatol Venereol* (2000)
Female	16Y	Swedish/Norwegian	Generalized	Present	Extensive	Extensive	No	Familial (no. 16.17)	Single heterozygous point	1A	Virtanen M et al., *Acta Derm Venereol* (2001)
Female	48Y	Swedish/Norwegian	Generalized	Present	Extensive	Mild	No	Familial (no. 15.17)	Single heterozygous point	1A	Virtanen M et al., *Acta Derm Venereol* (2001)
Female	74Y	Swedish/Norwegian	Generalized	Mild (feet)	Mild	Mild	No	Familial (no. 15.16)	Single heterozygous point	1A	Virtanen M et al., *Acta Derm Venereol* (2001)
Male	6Y	Swedish/Norwegian	EN	Present	Present	Streaks (lines of Blaschko)	No	Sporadic	Single heterozygous point	1A	Virtanen M et al., *Acta Derm Venereol* (2001)
Male	1Y	Chinese	Generalized	Mild (limbs)	Mild	Mild	No	Sporadic	Single heterozygous point	1A	Sun XK et al., *J Dermatol Sci* (2002)
Female	13Y	Chinese	Generalized	Present	Present	Present (limbs)	No	Sporadic	Single heterozygous point	1A	Zhang et al., *Chin J Med Genet* (2011)
NM	NM	German	Generalized	Diffuse	NM	Present	No	Sporadic	Single heterozygous point	1A	Arin MJ et al., *Br J Dermatol* (2011)
NM	NM	German	Generalized	Present	Present	Present (skin folds)	No	Familial	Single heterozygous point	1A	Arin MJ et al., *Br J Dermatol* (2011)
NM	NM	German	Generalized	Present	Present	Present (skin folds)	No	Sporadic	Single heterozygous point	1A	Arin MJ et al., *Br J Dermatol* (2011)
NM	NM	German	Generalized	Present	NM	Present (skin folds)	No	Sporadic	Single heterozygous point	1A	Arin MJ et al., *Br J Dermatol* (2011)
NM	NM	Polish	Generalized	Present	Present	NM	No	Familial^a^	Single heterozygous point	1A	Arin MJ et al., *Br J Dermatol* (2011)
NM	NM	German	Generalized	Present	NM	Present (skin folds)	No	Sporadic	Single heterozygous point	1A	Arin MJ et al., *Br J Dermatol* (2011)
NM	NM	German	Generalized	Diffuse	NM	NM	No	Familial	Single heterozygous point	1A	Arin MJ et al., *Br J Dermatol* (2011)
NM	NM	German	Generalized	Present	NM	Present (skin folds)	No	Sporadic	Single heterozygous point	1A	Arin MJ et al., *Br J Dermatol* (2011)
NM	NM	German	Generalized	Diffuse	NM	Present (skin folds)	No	Familial	Single heterozygous point	1A	Arin MJ et al., *Br J Dermatol* (2011)
NM	NM	Macedonian	Generalized	Present	Present	Present (knees, elbows)	No	Sporadic	Single heterozygous point	1A	Arin MJ et al., *Br J Dermatol* (2011)
NM	NM	German	Generalized	Present	NM	Present (skin folds)	No	Sporadic	Single heterozygous point	1A	Arin MJ et al., *Br J Dermatol* (2011)
NM	NM	German	EN	NM	NM	Streaks (axilla, trunk, leg)	No	Sporadic	Single heterozygous point	1A	Arin MJ et al., *Br J Dermatol* (2011)
Female	41Y	Chinese	Generalized	Present	Present	Extensive (entire body)	Desquamation	Familial	Single heterozygous point	1A	Li Z et al., *Ther Clin Risk Manag* (2014)
Male	13Y	Chinese	Generalized	Present	Extensive	Present	NM	Familial (child of 40)	Single heterozygous point	1A	Li Z et al., *Ther Clin Risk Manag* (2014)
Female	34Y	NM	Generalized	Mild	Present	Mild	NM	Familial	Single heterozygous point	1A	Hotz A et al., *Acta Derm Venereol* (2016)
Male	2Y	NM	Generalized	Present	Present	Present	NM	Familial (child of 42)	Single heterozygous point	1A	Hotz A et al., *Acta Derm Venereol* (2016)
Female	30Y	NM	Generalized	Present	Present	Present	NM	Sporadic	Single heterozygous point	1A	Hotz A et al., *Acta Derm Venereol* (2016)
Male	36Y	NM	Generalized	Present	Mild	Present	No	Sporadic	Single heterozygous point	1A	Hotz A et al., *Acta Derm Venereol* (2016)
Female	1Y	NM	Generalized	Present	Present	Present	NM	Sporadic	Single heterozygous point	1A	Hotz A et al., *Acta Derm Venereol* (2016)
Female	8Y	NM	Generalized	Present	Present	Present	NM	Sporadic	Single heterozygous point	1A	Hotz A et al., *Acta Derm Venereol* (2016)
Male	3.5M	NM	Annular EI	Mild (genital, limbs)	NM	Mild (knees, elbows, folds)	No	Sporadic	Single heterozygous point	1A	Reolid A et al., *JAAD Case Rep* (2019)
NM	35Y	Czechs	Generalized	Present	Present	Present (flexural)	No	NM	Single heterozygous point	1A	Borská et al., *Orphanet J Rare Dis* (2019)
NM	27Y	Czechs	Generalized	NM	NM	Present	NM	NM	Single heterozygous point	1A	Borská et al., *Orphanet J Rare Dis* (2019)
Female	9Y	Italian	Generalized	Present	Present	Extensive (joints)	No	Sporadic	Single heterozygous point	1A	Diociaiuti et al., *Int J Mol Sci* (2020)
Female	40Y	Italian	Generalized	Present (limbs)	Present	Extensive	No	Sporadic	Single heterozygous point	1A	Diociaiuti et al., *Int J Mol Sci* (2020)
Female	2Y	Chinese	Generalized	Present	Present	Present	No	Sporadic	Single heterozygous point	1A	Gan et al., *J Diagn Ther Dermato‐Venereology* (2022)
Male	8M	Chinese	Generalized	Mild	No	Mild (neck, flexural)	No	Sporadic	Single heterozygous point	1A	Yang Z et al., *Pediatr Investig* (2023)
Male	20M	Chinese	Generalized	Diffuse	Mild	Extensive (joints, neck, flexural)	No	Sporadic	Single heterozygous point	1A	Yang Z et al., *Pediatr Investig* (2023)
Male	NM	Chinese	EN	NM	NM	Extensive streaks (lines of Blaschko)	NM	Sporadic	Single heterozygous point	1A	Song D et al., *Int J Dermatol* (2023)
Female	2M	Chinese	Generalized	Present (trunk, limbs)	Extensive	NM	No	Familial^a^ (child of 56)	Single heterozygous point	1A	Song D et al., *Int J Dermatol* (2023)
Female	39Y	NM	Generalized	Absent	Extensive	Extensive (face, trunk, extremities)	No	Sporadic	Single heterozygous point	1A	Phusuphitchayanan P et al., *Health Sci Med Res* (2023)

*Note:* Also minor types of EI, with this specific mutation, are mentioned. Open access of the article was required for inclusion in the table.

Abbreviations: EI, epidermolytic ichthyosis; EN, epidermolytic nevi; M, months old; NM, not mentioned in published report; PPK, palmoplantar keratoderma; Y, years old.

^a^ Paternal cutaneous‐gonadal mosaicism (transmission of the KRT10 mutation from mosaic to germline).

Neonatal bullous skin problems can be divided into infectious diseases (SSSS, herpes, varicella, candida), toxicoderma (toxic epidermal necrolysis), immunodeficiency disease (SCID), autoimmune diseases (neonatal pemphigoid, neonatal pemphigus), histiocytosis, mastocytosis, and genetic skin disorders (particularly EB and EI). Among inherited blistering conditions in neonates, EB is usually the most commonly diagnosed. The phenotypes of EB and EI can be hard to distinguish, as both present with blister formation as a result of disturbances in the keratin filament networks [18]. EI differs from EB in that suprabasal rather than basal cells of the epidermis are prone to cytolysis, so the basal layer remains intact [10]. Anatomopathology and immunofluorescence antigen mapping will lead to a diagnosis of EB, but will be negative or nonspecific for EI. An overview of the main characteristics between these two diseases is presented in Table 2.

**TABLE 2:** Overview of features between epidermolytic hyperkeratosis and epidermolysis bullosa (inherited bullous disorders).

	Epidermolytic ichthyosis [1]	Epidermolysis bullosa			
		EB simplex [19]	Junctional EB [19]	Dystrophic EB [19]	Kindler syndrome [20]
Inheritance pattern	AD	AD	AR	AD or AR	AR
Genes affected	KRT1, KRT10	KRT5, KRT14	LAMA3, LAMB3, LAMC2	COL7A1	FERMT1
Cytogenetic location [21]	17q21‐q23	12q13.13	18q11.2	3p21.31	20p12.3
Encoding proteins	Keratine 1, keratine 10	Keratine 5, keratine 14	Laminin 332	Type VII collagen	Kindlin‐1
Prevalence	1:200000	70% of EB cases (1:100000) [22, 23]	5% of EB cases (1:100000) [22, 23]	25% of EB cases (1:100000) [22, 23]	Around 250 cases worldwide
Onset	At birth	At birth	At birth	At birth	At birth
Clinical findings	Erythroderma, blistering, ichthyotic scaling	Blistering, healing without scarring	Blistering, nail and dental defects, airway involvement	Blistering, scarring of skin and mucous membranes	Blistering, poikiloderma, photosensitivity, mucosal involvement
Level of cytolysis	Suprabasal layers of epidermis	Basal layers of epidermis	Dermal‐epidermal junction	Upper dermis	Mixed types

*Note:* Adapted from Peter Rout et al. [1], Fine [19], Has et al. [20], Orphanet [21], Bruckner et al. [22], and Fine [23].

Abbreviations: AD, autosomal dominant; AR, autosomal recessive; EB, epidermolysis bullosa.

The initial clinical presentation in this patient was challenging, characterized by extensive areas of erosive and denuded skin, with prolonged persistence of bullous and erosive lesions (until at least 14 months of age, the time of the last follow‐up). The concurrent hematologic, renal, and pulmonary abnormalities further complicated the clinical picture, prompting consideration of a broader differential diagnosis. These systemic issues, however, resolved spontaneously. To our knowledge, such complications have not been previously reported in cases of EI. The transient renal impairment was likely secondary to the use of nephrotoxic medication and dehydration resulting from significant transepidermal fluid loss. Additional research is necessary to determine the potential impact of *KRT10* pathogenic variants on pulmonary function [24]. Extracutaneous anomalies, including ectodermal malformations such as microtia and dorsal acral hypertrichosis, have been reported in several studies and are more commonly linked to *KRT10* mutations than to *KRT1* mutations [25, 26, 27, 28]. Different expression sites of keratin 1 and keratin 10 may result in this distinct clinical presentation. These findings underscore the importance of comprehensive clinical evaluation in affected neonates and highlight the need for further investigation into the prenatal expression of *KRT10* in humans.

Different clinical presentations of EI are described, which can be divided into distinct groups with presence or absence of severe palmoplantar keratoderma (PPK). PPK, where palms of the hands and soles of the feet are involved, often leads to recurrent painful fissures and contractures that can diminish the mobility of joints. *KRT1* pathogenic variants are associated with PPK, and *KRT10* pathogenic variants are associated with milder or no palmoplantar involvement [29]. But exceptions are reported for where *KRT10* pathogenic variants caused severe EI with palmoplantar involvement [30, 31, 32]. The presented case makes no exception to the general rule, with no palmoplantar keratosis observed at the time of follow‐up. Blistering that becomes less frequent and is replaced by hyperkeratotic plaques and scaling is the known natural evolution of EI after the first months of life. The hyperkeratosis is mainly located on flexural and intertriginous regions, as our reported case confirms, and gives a characteristic cobblestone appearance. It may also occur on the scalp and neck.

Management of EI is primarily focused on pain relief and symptom control, as no cure is available. Extensive wound care with appropriate wound dressing is paramount to provide timely wound healing and prevent infections. Adequate, multimodal analgesia during wound care and in between dressing changes should be pursued. When a congenital blistering skin disease is suspected in a newborn, prompt transfer to a well‐equipped facility with the necessary knowledge is advised, as a multidisciplinary team approach should be followed. Family support is another major pillar of the management. Support for parents is essential, as the diagnosis of EI can be overwhelming and stressful. Inclusion of parents and caregivers in the daily care of the infant and education about close monitoring of infection are important. Prior to discharge, follow‐up appointments with the multidisciplinary team should be planned. Skin evolution, psychological wellbeing, pain control, growth, and thriving should be monitored.

This case underscores the variability and clinical complexity of EI, emphasizing the importance of heightened clinical awareness, a broad differential diagnosis, and a multidisciplinary approach to ensure optimal patient care. Genetic testing plays a crucial role in the early diagnosis of neonatal blistering disorders.

## Author Contributions

**Elke Smits:** conceptualization, investigation, project administration, visualization, writing – original draft. **Gunnar Naulaers:** conceptualization, investigation, resources, supervision, writing – review and editing. **Maria C. Bolling:** visualization, writing – review and editing. **Eric Legius:** writing – review and editing. **Caroline Colmant:** writing – review and editing.

## Ethics Statement

This study was approved by the Ethics Committee Research at the University Hospital of Leuven, Belgium, and performed in line with the principles of the Declaration of Helsinki.

## Consent

Written informed consent was obtained from the patient's guardian for publication of the case and associated clinical images. The guardian has been fully informed about the nature of the publication and has agreed to the use of the patient's information.

## Conflicts of Interest

The authors declare no conflicts of interest.

## Data Availability

The authors have nothing to report.

## References

[1] D. Peter Rout , A. Nair , A. Gupta , and P. Kumar , “Epidermolytic Hyperkeratosis: Clinical Update,” Clinical, Cosmetic and Investigational Dermatology 12 (2019): 333–344. 31190940 10.2147/CCID.S166849 PMC6512611

[2] T. Takeichi and M. Akiyama , “Inherited Ichthyosis: Non‐Syndromic Forms,” Journal of Dermatology 43, no. 3 (2016): 242–251, 10.1111/1346-8138.13243. 26945532

[3] N. L. Lacz , R. A. Schwartz , and G. Kihiczak , “Epidermolytic Hyperkeratosis: A Keratin 1 or 10 Mutational Event,” International Journal of Dermatology 44, no. 1 (2005): 1–6. 10.1111/j.1365-4632.2004.02364.x 15663649

[4] R. Ross , J. J. Digiovanna , L. Capaldi , Z. Argenyi , P. Fleckman , and L. Robinson‐bostom , “Histopathologic Characterization of Epidermolytic Hyperkeratosis: A Systematic Review of Histology From the National Registry for Ichthyosis and Related Skin Disorders,” Journal of the American Academy of Dermatology 2008, no. 59 (2008): 86–90. 10.1016/j.jaad.2008.02.031 PMC2517215 18571597

[5] M. Avril , “Management of Epidermolytic Ichthyosis in the Newborn,” Neonatal Network: NN 35, no. 1 (2016): 19–29. 26842536 10.1891/0730-0832.35.1.19

[6] J. Cheng , A. J. Syder , Q. C. Yu , A. Letal , A. S. Paller , and E. Fuchs , “The Genetic Basis of Epidermolytic Hyperkeratosis: A Disorder of Differentiation‐Specific Epidermal Keratin Genes,” Cell 70, no. 5 (1992): 811–819, 10.1016/0092-8674(92)90314-3. 1381287

[7] Z. S. De , J. J. Liu , W. Tian , Z. J. Zhao , and J. J. Zhao , “Mutation Analysis of KRT10 Gene in a Patient With Bullous Congenital Ichthyosiform Erythroderma,” Zhonghua Yi Xue Yi Chuan Xue Za Zhi = Zhonghua Yixue Yichuanxue Zazhi = Chinese Journal of Medical Genetics 28, no. 4 (2011): 421–423, 10.3760/cma.j.issn.1003-9406.2011.04.014. 21811984

[8] K. Nomura , X. Meng , K. Umeki , et al., “A Keratin K10 Gene Mutation in a Japanese Patient With Epidermolytic Hyperkeratosis,” Japanese Journal of Human Genetics 42, no. 1 (1997): 217–223, 10.1007/BF02766925. 9184002

[9] N. Mayuzumi , T. Shigihara , S. Ikeda , and H. Ogawa , “Recurrent R156H Mutation of KRT10 in a Japanese Family With Bullous Congenital Ichthyosiform Erythroderma,” Journal of the European Academy of Dermatology and Venereology 14, no. 4 (2000): 304–306, 10.1046/j.1468-3083.2000.00101.x. 11204523

[10] E. Fuchs , R. A. Esteves , and P. A. Coulombet , “Transgenic Mice Expressing a Mutant Keratin 10 Gene Reveal the Likely Genetic Basis for Epidermolytic Hyperkeratosis,” Proceedings of the National Academy of Sciences of the United States of America 89 (1992): 6906–6910, 10.1073/pnas.89.15.6906. 1379726 PMC49613

[11] R. B. Mary , A. Longley , D. S. Bundman , et al., “A Transgenic Mouse Model That Recapitulates the Clinical Features of Both Neonatal and Adult Forms of the Skin Disease Epidermolytic Hyperkeratosis,” Differentiation 61, no. 2 (1996): 129–139, 10.1046/j.1432-0436.1996.6120129.x. 8983179

[12] O. P. March , T. Lettner , A. Klausegger , et al., “Gene Editing–Mediated Disruption of Epidermolytic Ichthyosis–Associated KRT10 Alleles Restores Filament Stability in Keratinocytes,” Journal of Investigative Dermatology 139, no. 8 (2019): 1699–1710, 10.1016/j.jid.2019.03.1146. 30998984

[13] A. Vahlquist , J. Fischer , and H. Törmä , “Inherited Nonsyndromic Ichthyoses: An Update on Pathophysiology, Diagnosis and Treatment,” American Journal of Clinical Dermatology 19, no. 1 (2018): 51–66, 10.1007/s40257-017-0313-x. 28815464 PMC5797567

[14] S. M. Paramio , M. L. Casanova , C. Segrelles , S. Mittnacht , and E. B. Lane , “Modulation of Cell Proliferation by Cytokeratins K10 and K16,” Molecular and Cellular Biology 19, no. 4 (1999): 3086–3094. 10082575 10.1128/mcb.19.4.3086 PMC84102

[15] V. Oji , G. Tadini , M. Akiyama , et al., “Revised Nomenclature and Classification of Inherited Ichthyoses: Results of the First Ichthyosis Consensus Conference in Sorze 2009,” Journal of the American Academy of Dermatology 63, no. 4 (2010): 607–641, 10.1016/j.jaad.2009.11.020. 20643494

[16] K. Haruna , Y. Suga , Y. Mizuno , et al., “R156C Mutation of Keratin 10 Causes Mild Form of Epidermolytic Hyperkeratosis,” Journal of Dermatology 34, no. 8 (2007): 545–548, 10.1111/j.1346-8138.2007.00328.x. 17683385

[17] A. Abdul‐Wahab , T. Takeichi , L. Liu , C. Stephens , M. Akiyama , and J. A. McGrath , “Intrafamilial Phenotypic Heterogeneity of Epidermolytic Ichthyosis Associated With a New Missense Mutation in Keratin 10,” Clinical and Experimental Dermatology 41, no. 3 (2016): 290–293, 10.1111/ced.12751. 26338057

[18] E. Fuchs , P. Coulombe , J. Cheng , et al., “Genetic Bases of Epidermolysis Bullosa Simplex and Epidermolytic Hyperkeratosis,” Journal of Investigative Dermatology 103 (1994): S25–S30. 10.1111/1523-1747.ep12398924 7525738

[19] J. D. Fine , “Inherited Epidermolysis Bullosa,” Orphanet Journal of Rare Diseases 5 (2010): 12, 10.1186/1750-1172-5-12. 20507631 PMC2892432

[20] C. Has , J. W. Bauer , C. Bodemer , et al., “Consensus Reclassification of Inherited Epidermolysis Bullosa and Other Disorders With Skin Fragility,” British Journal of Dermatology 183, no. 4 (2020): 614–627, 10.1111/bjd.18921. 32017015

[21] “Orphanet: An Online Rare Disease and Orphan Drug Data Base,” Copyright, INSERM 1999, 2024, https://www.orpha.net.

[22] A. L. Bruckner , M. Losow , J. Wisk , et al., “The Challenges of Living With and Managing Epidermolysis Bullosa: Insights From Patients and Caregivers,” Orphanet Journal of Rare Diseases 15, no. 1 (2020): 1, 10.1186/s13023-019-1279-y. 31900176 PMC6942340

[23] J. D. Fine , “Epidemiology of Inherited Epidermolysis Bullosa Based on Incidence and Prevalence Estimates From the National Epidermolysis Bullosa Registry,” JAMA Dermatology 152, no. 11 (2016): 1231–1238, 10.1001/jamadermatol.2016.2473. 27463098

[24] P. Shivshankar , C. Sanchez , L. F. Rose , and C. J. Orihuela , “The *Streptococcus pneumoniae* Adhesin PsrP Binds to Keratin 10 on Lung Cells,” Molecular Microbiology 73, no. 4 (2009): 663–679, 10.1111/j.1365-2958.2009.06796.x. 19627498 PMC2753542

[25] I. Spoerri , M. Brena , J. De Mesmaeker , et al., “The Phenotypic and Genotypic Spectra of Ichthyosis With Confetti Plus Novel Genetic Variation in the 3′ End of KRT10,” JAMA Dermatology 151, no. 1 (2015): 64–69, 10.1001/jamadermatol.2014.2526. 25210931

[26] A. Hotz , V. Oji , E. Bourrat , et al., “Expanding the Clinical and Genetic Spectrum of KRT1, KRT2 and KRT10 Mutations in Keratinopathic Ichthyosis,” Acta Dermato‐Venereologica 96, no. 4 (2016): 473–478, 10.2340/00015555-2299. 26581228

[27] S. H. Foo , A. Terron‐Kwiatkowski , D. Baty , and F. Browne , “Ichthyosis With Confetti Presenting as Collodion Baby: A Novel Mutation in KRT10,” Clinical and Experimental Dermatology 42, no. 5 (2017): 543–544, 10.1111/ced.13097. 28556375

[28] B. Kurz , K. T. Koschitzki , U. Hehr , et al., “Congenital Ichthyosiform Erythroderma With Epidermolysis due to a Novel Frameshift Mutation in KRT10,” JAAD Case Reports 35 (2023): 74–76, 10.1016/j.jdcr.2023.02.028. 37101807 PMC10123060

[29] J. Digiovanna and S. J. Bale , “Clinical Heterogeneity in Epidermolytic Hyperkeratosis,” Archives of Dermatology 130 (1994): 1026–1035. 8053700

[30] M. Virtanen , S. K. Smith , T. Gedde‐Dahl, Jr. , A. Vahlquist , and P. E. Bowden , “Splice Site and Deletion Mutations in Keratin (KRT1 and KRT10) Genes: Unusual Phenotypic Alterations in Scandinavian Patients With Epidermolytic Hyperkeratosis,” Journal of Investigative Dermatology 121, no. 5 (2003): 1013–1020, 10.1046/j.1523-1747.2003.12534.x. 14708600

[31] M. J. Arin , M. A. Longley , I. Anton‐lamprecht , et al., “A Novel Substitution in Keratin 10 in Epidermolytic Hyperkeratosis,” Journal of Investigative Dermatology 112, no. 4 (1999): 506–508, 10.1046/j.1523-1747.1999.00557.x. 10201536

[32] W. H. I. McLean , S. M. Morley , C. Higgins , et al., “Novel and Recurrent Mutations in Keratin 10 Causing Bullous Congenital Ichthyosiform Erythroderma,” Experimental Dermatology 8, no. 2 (1999): 120–123, 10.1111/j.1600-0625.1999.tb00358.x. 10232402

